# Tumor microenvironment conditions alter Akt and Na^+^/H^+^ exchanger NHE1 expression in endothelial cells more than hypoxia alone: implications for endothelial cell function in cancer

**DOI:** 10.1186/s12885-017-3532-x

**Published:** 2017-08-14

**Authors:** A. K. Pedersen, J. Mendes Lopes de Melo, N. Mørup, K. Tritsaris, S. F. Pedersen

**Affiliations:** 1 Department of Cellular and Molecular Medicine, Faculty of Health and Medical Sciences, University of Copenhagen, Panum Institute, Blegdamsvej 3B, 2200 Copenhagen, Denmark; 2 Section for Cell Biology and Physiology, Department of Biology, Faculty of Science, University of Copenhagen, Universitetsparken 13, 2100 Copenhagen, Denmark

**Keywords:** Cancer, Angiogenesis, VEGF, Signaling, Acid–base transport, pH regulation, Proliferation

## Abstract

**Background:**

Chronic angiogenesis is a hallmark of most tumors and takes place in a hostile tumor microenvironment (TME) characterized by hypoxia, low nutrient and glucose levels, elevated lactate and low pH. Despite this, most studies addressing angiogenic signaling use hypoxia as a proxy for tumor conditions. Here, we compared the effects of hypoxia and TME conditions on regulation of the Na^+^/H^+^ exchanger NHE1, Ser/Thr kinases Akt1–3, and downstream effectors in endothelial cells.

**Methods:**

Human umbilical vein endothelial cells (HUVEC) and Ea.hy926 endothelial cells were exposed to simulated TME (1% hypoxia, low serum, glucose, pH, high lactate) or 1% hypoxia for 24 or 48 h, with or without NHE1 inhibition or siRNA-mediated knockdown. mRNA and protein levels of NHE1, Akt1–3, and downstream effectors were assessed by qPCR and Western blotting, vascular endothelial growth factor (VEGF) release by ELISA, and motility by scratch assay.

**Results:**

Within 24 h, HIF-1α level and VEGF mRNA level were increased robustly by TME and modestly by hypoxia alone. The NHE1 mRNA level was decreased by both hypoxia and TME, and NHE1 protein was reduced by TME in Ea.hy926 cells. Akt1–3 mRNA was detected in HUVEC and Ea.hy926 cells, Akt1 most abundantly. Akt1 protein expression was reduced by TME yet unaffected by hypoxia, while Akt phosphorylation was increased by TME. The Akt loss was partly reversed by MCF-7 human breast cancer cell conditioned medium, suggesting that in vivo, the cancer cell secretome may compensate for adverse effects of TME on endothelial cells. TME, yet not hypoxia, reduced p70S6 kinase activity and ribosomal protein S6 phosphorylation and increased eIF2α phosphorylation, consistent with inhibition of protein translation. Finally, TME reduced Retinoblastoma protein phosphorylation and induced poly-ADP-ribose polymerase (PARP) cleavage consistent with inhibition of proliferation and induction of apoptosis. NHE1 knockdown, mimicking the effect of TME on NHE1 expression, reduced Ea.hy926 migration. TME effects on HIF-1α, VEGF, Akt, translation, proliferation or apoptosis markers were unaffected by NHE1 knockdown/inhibition.

**Conclusions:**

NHE1 and Akt are downregulated by TME conditions, more potently than by hypoxia alone. This inhibits endothelial cell migration and growth in a manner likely modulated by the cancer cell secretome.

**Electronic supplementary material:**

The online version of this article (doi:10.1186/s12885-017-3532-x) contains supplementary material, which is available to authorized users.

## Background

Chronic angiogenesis is a hallmark of most cancers. With the possible exception of a few “hypovascular” cancers such as pancreatic adenocarcinomas, the “angiogenic switch” – the onset of tumor angiogenesis – is essential for continued tumor growth [1–3]. Accordingly, angiogenesis inhibitors have shown efficacy in restricting both primary tumor growth and metastasis in many types of cancers in preclinical models. Several anti-angiogenic compounds are in clinical use and are initially effective, yet generally fail to produce a lasting response [1–4].

Hypoxic conditions arise as soon as the tumor grows beyond a few hundred μm in diameter. Hypoxia increases the protein level and activity of the transcription factor Hypoxia-Inducible Factor 1α (HIF-1α), in turn inducing the upregulation of Vascular Endothelial Growth Factor (VEGF) A and VEGF receptor 2 (VEGFR2) in the endothelial cells, resulting in endothelial cell proliferation and angiogenesis [5, 6]. In tumors, the cancer cells are a major source of secreted VEGF, further stimulating angiogenesis [2]. In congruence with the central role of VEGF, the humanized, VEGF-neutralizing monoclonal antibody bevacizumab is approved for treatment of several cancers, including late-stage colon cancer and breast cancer, in conjunction with chemotherapy [1, 7].

In addition to being hypoxic, the tumor microenvironment (TME) is characterized by acidic extracellular pH (pH_e_), low glucose and nutrient levels, elevated lactate levels, and the presence of multiple cytokines and growth factors, including VEGF, secreted by the various cell types present in the tumor [8]. Notably, while hypoxia in isolation is often taken as relevant to cancer biology, other chemico-physical conditions of the TME (hypoxia, acidic pH, low glucose, high lactate) can exert profoundly different gene-regulatory effects than hypoxia alone [9–12], yet have been much less studied. The dysregulation of pH_e_ results from increased metabolic acid production in, and net acid extrusion from, the cancer cells, in conjunction with poor diffusion in the TME. This results in pH_e_ as low as 6.2–6.5 [13, 14]. The increased acid extrusion from the cancer cells reflects the upregulation and increased activity of several acid–base transport proteins, including the Na^+^/H^+^ exchanger NHE1 [13–15]. NHE1 has been shown to contribute to increased motility, invasiveness, proliferation and survival in a wide range of cancer cell types [13–18]. Furthermore, NHE1 was recently assigned a role in VEGF secretion from KN562 leukemia cells [19, 20]. In contrast, the role of NHE1 in tumor endothelial cells is essentially unknown. NHE1 is expressed in various types of endothelial cells and contributes to their intracellular pH (pH_i_) regulation and consequently to endothelial function [21–24]. NHE1 was reported to be upregulated by exogenous expression of HIF-1α in Human Umbilical Vein Endothelial Cells (HUVEC) [25], and its pharmacological inhibition or knockdown was found to attenuate HIF-1α-induced HUVEC proliferation, migration and tube formation [25]. Furthermore, NHE1 expression and activity were upregulated by hypoxia, aglycemia, or vasopressin in blood–brain barrier endothelial cells [24]. In non-endothelial cells, the impact of hypoxia on NHE1 is controversial. Initial reports found NHE1 expression to be increased in a HIF-1α-dependent manner in pulmonary arterial myocytes [26], whereas a later report in a wide array of cancer cell lines found NHE1 expression to be either downregulated or unaffected by hypoxia [27].

The Ser/Thr kinase Akt, acting downstream from activation of VEGFR2, plays central roles in regulation of endothelial cell function, including the control of vessel growth and homeostasis [5, 28, 29]*.* Three closely related Akt isoforms, Akt1–3, are expressed in mammalian cells, Akt1 being the most abundant and widely expressed. The three isoforms are structurally similar, yet exhibit functional differences in several cell types including endothelial cells [30–32]. Akt is an important regulator of cell growth, in part via its ability to stimulate the phosphorylation of the p70S6 kinase (p70S6K), leading to ribosomal protein S6 (rpS6) phosphorylation [33]. Notably, in non-endothelial cells, NHE1 has been shown to recruit and activate Akt [34] and, conversely, to be phosphorylated by Akt suggesting that these two important regulators of endothelial function might be functionally linked.

Thus, NHE1 and Akt are important for endothelial cell function, and are regulated, directly or indirectly, by hypoxia. However, the impact of hypoxia on NHE1 is controversial, and the impact of the more complex physicochemical TME on NHE1 and Akt in endothelial cells has, to our knowledge, never been studied. Here, we compared the effect of hypoxia alone to that of TME on NHE1 and Akt1–3 in primary endothelial cells and an endothelial cell line, and assessed the effect of pharmacological and siRNA-mediated NHE1 inhibition on Akt expression, activity, and endothelial cell function. We report that NHE1, Akt, and protein translation signaling are downregulated much more potently by TME conditions than by hypoxia alone, and that this inhibits endothelial cell migration, proliferation and survival, in a manner likely to be modulated by the cancer cell secretome.

## Methods

### Cell lines and culture conditions

Primary human umbilical vein endothelial cells (HUVEC, [35]) from pooled donors (Lonza, CC-2519) were cultured in gelatine-coated cell culture flasks in EBM basal medium (Lonza) supplemented with EGM singleQuot supplement and growth factors (Lonza). Cells were maintained at 37 °C under 5% CO_2_ and 95% humidity and experiments were performed with cells in passage 4–9. The hybrid EA.hy926 cell line, generated by fusion of HUVEC with cells of the lung carcinoma cell line A549 [36], was cultured in 1% gelatine-coated cell culture flasks in DMEM 1965 medium supplemented with 10% fetal bovine serum (FBS) and 1% penicillin/streptomycin. Cells were maintained like HUVEC and not used above passage 20.

### Cell culture and model system

Under experimental conditions, cells were grown in RPMI-1640 (Sigma-Aldrich). For control conditions RPMI-1640 was supplemented with 5% FBS, 10 mM glucose, 5 mM NaCl, 1% penicillin/streptomycin and 24 mM HCO_3_^−^ to reach a pH of 7.4 when equilibrating with 5% CO_2_ (control (ctrl) medium). To mimic tumor microenvironment (TME) conditions RPMI-1640 was supplemented with 1% FBS, 2.5 mM glucose, 10 mM NaCl, 7.5 mM Sodium Lactate (NaL), 1% penicillin/streptomycin and 3 mM HCO_3_^−^ to equilibrate to a pH of 6.5 when incubated with 5% CO_2_ (TME medium). For experiments, cells were grown in 1% gelatine-coated dishes in regular growth medium, washed with PBS and incubated with either control or TME medium for 24 or 48 h. Control cells were kept at 37 °C with 5% CO_2_ and 95% humidity. Cells in TME medium were incubated in a computer-controlled workspace/incubator system (Xvivo G300C, Biospherix) at 37 °C with 5% CO_2_ and 94% humidified Nitrogen (N_2_) and 1% O_2_, essentially as previously described [37]. For hypoxia alone, cells in control medium were exposed to 5% CO_2_ and 94% N_2_ and 1% O_2_ as described for the TME cells. For functional inhibition of NHE1, cells were treated with 10 μM cariporide (a kind gift from Sanofi Aventis). Cariporide was dissolved at 10 mM in ddH_2_O. MCF-7 human breast cancer cells (a kind gift from Dr. Lone Ronnov-Jessen, University of Copenhagen) were grown in standard low glucose (5.5 mM) DMEM 1885 (SSC, University of Copenhagen, Cat. 22–2-24, #015) supplemented with 6% FBS (Gibco), 1% Pen/Strep (Invitrogen), and 1% MEM Non-Essential Amino Acids 100X (Gibco/Invitrogen), at 37 °C, 95% humidity, 5% CO_2_. For TME experiments, cells were seeded in culture dishes and exposed to TME medium as above for 24 or 48 h.

### SDS-PAGE and western blotting

For western blotting, cells were washed in ice-cold PBS and lysed with SDS lysis buffer (1% SDS, 10 mM Tris–HCl, 1 mM Na_3_VO_4_, pH 7.5). The cell lysate was homogenized by sonication and centrifuged for 10 min at 13,000 g. Protein contents were quantified using the Bio-Rad DC protein assay kit (Bio-Rad) and samples were diluted to equal protein concentrations, with water and 1X loading buffer (Invitrogen), containing dithiothretiol (DTT) (Sigma-Aldrich). Proteins were separated by SDS-PAGE gel electrophoresis (NuPAGE 4–12% Bis-Tris gels) under denaturing and reducing conditions (NuPAGE MES SDS running buffer) (Invitrogen). Proteins were transferred onto nitrocellulose membranes with transfer buffer containing 20% methanol. Membranes were blocked in 5% milk protein in PBS-Tween (1xPBS with 0.1% Tween-20) for 2 h at room temperature and incubated with primary antibody diluted in PBS-Tween with 5% BSA and 0,1% NaN_3_ overnight at 4 °C. Primary antibodies used were rabbit anti-phospho(p)-Ser473Akt (#9271), rabbit anti-Akt1 (#2962), rabbit anti-p-Ser51 eIF2α (#9721), rabbit anti-GAPDH (#2118), mouse anti-pThr389-p70S6K (#9206), rabbit anti-p70S6K (#2708), rabbit anti-poly-ADP-ribose polymerase (PARP) (#9542), rabbit anti-p-Ser807/811-pRb (#9308), and rabbit anti-p-Ser235/236-rpS6 (#4856) purchased from Cell Signaling Technology; mouse anti-p-Thr202/Tyr204-ERK1/2 (#7383) and mouse anti-NHE1 (clone 54) purchased from Santa Cruz Biotechnology; mouse anti-GAPDH (CB1001) purchased from Millipore; mouse anti-HIF-1α (#610958) and mouse anti-p150 (#610473) purchased from BD Transduction Laboratories. Membranes were incubated with horseradish peroxidase-conjugated secondary antibody (Dako; goat-anti-mouse IgG, #P0447, goat-anti-rabbit IgG, #P 0448) diluted in 1% milk protein in PBS-Tween for 1 h at room temperature and developed with Supersignal West Pico chemiluminescent substrate or Supersignal West Femto maximum sensitivity substrate (Thermo Fisher Scientific) using the UVP Biospectrum chemiluminescence Imaging system. Images were obtained using the VisionWorksLS software and UN-SCAN-IT 6.1 (Silk Scientific) was utilized to quantify the intensity of the protein bands.

### qPCR analysis

Total RNA was purified using the RNeasy mini kit (Qiagen) and the RNase free DNase set (Qiagen), and reverse transcription was performed using the Omniscript RT mini kit (Qiagen), all according to the instructions of the manufacturer. cDNA was amplified by quantitative PCR using LightCycler 480 SYBR Green I Master Mix (Roche Applied Sciences), according to the instructions of the manufacturer. Reactions were carried out in triplicates on a Stratagene Mx3005P QPCR system from Agilent Technologies (95 °C 10 min, 40 cycles of 95 °C 20 s, annealing temperature 58–64 °C depending on primers 22 s and 72 °C 20 s). The following primer pairs were used: 5′-CTTTGCCGGTATCGTGTGGC-3′ (forward) and 5′-CTCGCTGTCCACACACTCCA-3′(reverse) to generate an Akt1 fragment of 172 bp; 5′-TCAAAGAAGGCTGGCTCCAC-3 (forward) and 5′-GGCCTCTCGGTCTTCATCAG-3 (reverse) to generate an Akt2 fragment of 184 bp; 5′-CACCACCTGAAAAATATGATGAGGA-3 (forward) and 5′-GGTGCCCCTGCTATGTGTAA-3 (reverse) to generate an Akt3 fragment of 200 bp; 5′-GGAAGGTGAAGGTCGGAGTCAA-3′(forward) and 5′-GATCTCGCTCCTGGAAGATGCAT-3′(reverse) to generate a GAPDH fragment of 240 bp; 5′-CACACCACCATCAAATACTTCC-3′(forward) and 5′-GAACTTGTTGATGAACCAGGTC-3′ to generate an NHE1 fragment of 192 bp; and 5′-GCGTTGCAAGGCGAGGCAGC-3′(forward) and 5′-TGGTGGCGGCAGCGTGGTTT-3′(reverse) to generate a VEGF fragment of 172 bp. Amplification of specific targets were verified by agarose-gel electrophoresis. A standard curve of 4× serial dilutions of cDNA was made for each of the utilized primer-sets and the Pfaffl method was applied for relative quantification of the qPCR results, using GAPDH as the reference gene.

### siRNA-mediated knockdown of NHE1

NHE1 siRNA (NM_003047; SASI_Hs01_00025997) and universal negative control siRNA (SIC001) were obtained from Sigma-Aldrich. For siRNA transfection, EA.hy926 cells were grown to 50–60% confluency in gelatine-coated culture dishes and transfected with NHE1 siRNA (50 nM) or scrambled siRNA (50 nM), using the N-TER nanoparticle siRNA transfection system (Sigma-Aldrich), according to the instructions of the manufacturer. For western blotting and qPCR cells were lysed 48 h post transfection.

### ELISA assay for VEGF release

VEGF concentration in conditioned medium was determined using the human VEGF DuoSet ELISA Development kit (R&D Systems), according to the instructions of the manufacturer. In short, the plate was coated with a capture antibody against VEGF followed by blocking of the wells with reagent diluent (PBS with 1% BSA). Next, samples were added to the plate and bound VEGF was detected with biotinylated detection antibody, followed by addition of streptavidin conjugated to HRP. In between each step the plate was washed 3 times with wash buffer (PBS with 0.05% Tween-20). Substrate solution (R&D Systems) was added to the plate, reaction was stopped with 2 N sulfuric acid (H_2_SO_4_) and the optical densities of the wells were determined at 450 nm on a Synergy HT microplate reader from BioTek, with wavelength correction set to 540 nm. The amount of VEGF in the samples was quantitatively determined according to a 7-point standard curve of 2× serial dilutions of known concentrations. Both standards and samples were measured in triplicates.

### Scratch assay

An in vitro scratch assay was performed with siNHE1-transfected EA.hy926 cells, essentially as described in [38]. In short, cells were grown to 50–60% confluency before transfection with NHE1 siRNA. Cells were then incubated for 48 h, where after a scratch was made in the cell monolayer and cell movement and wound closure were monitored at different time intervals. After scratch induction cells were washed with PBS, changed to fresh complete medium and left to adapt for approximately 6 h (during which no change was observed). Images of wound area used for quantification were acquired after adaptation (considered *t* = 0) and at *t* = 18 h. Visualization and image acquisition were performed using a Leitz Labovert phase-contrast microscope (Leica Microsystems) and a digital camera (CoolPix 990, Nikon).

### Statistical analysis

Results are presented as representative individual experiments or as mean values with error bars showing standard error of means (SEM). Statistical analysis was done with GraphPad Prism using either two-way or one-way analysis of variance (ANOVA) with Tukey’s or Bonferroni’s multiple comparison post-test, a one-sample *t*-test (Fig. 4a) or a two-tailed paired Students *t*-test, as indicated in the figure legends. Statistically significant results are marked with *, **, *** or **** denoting *p* < 0.05, *p* < 0.01, *p* < 0.001 or *p* < 0.0001, respectively.

## Results

### TME conditions upregulate HIF-1α more than hypoxia alone and independent of NHE1

**Fig. 1:**
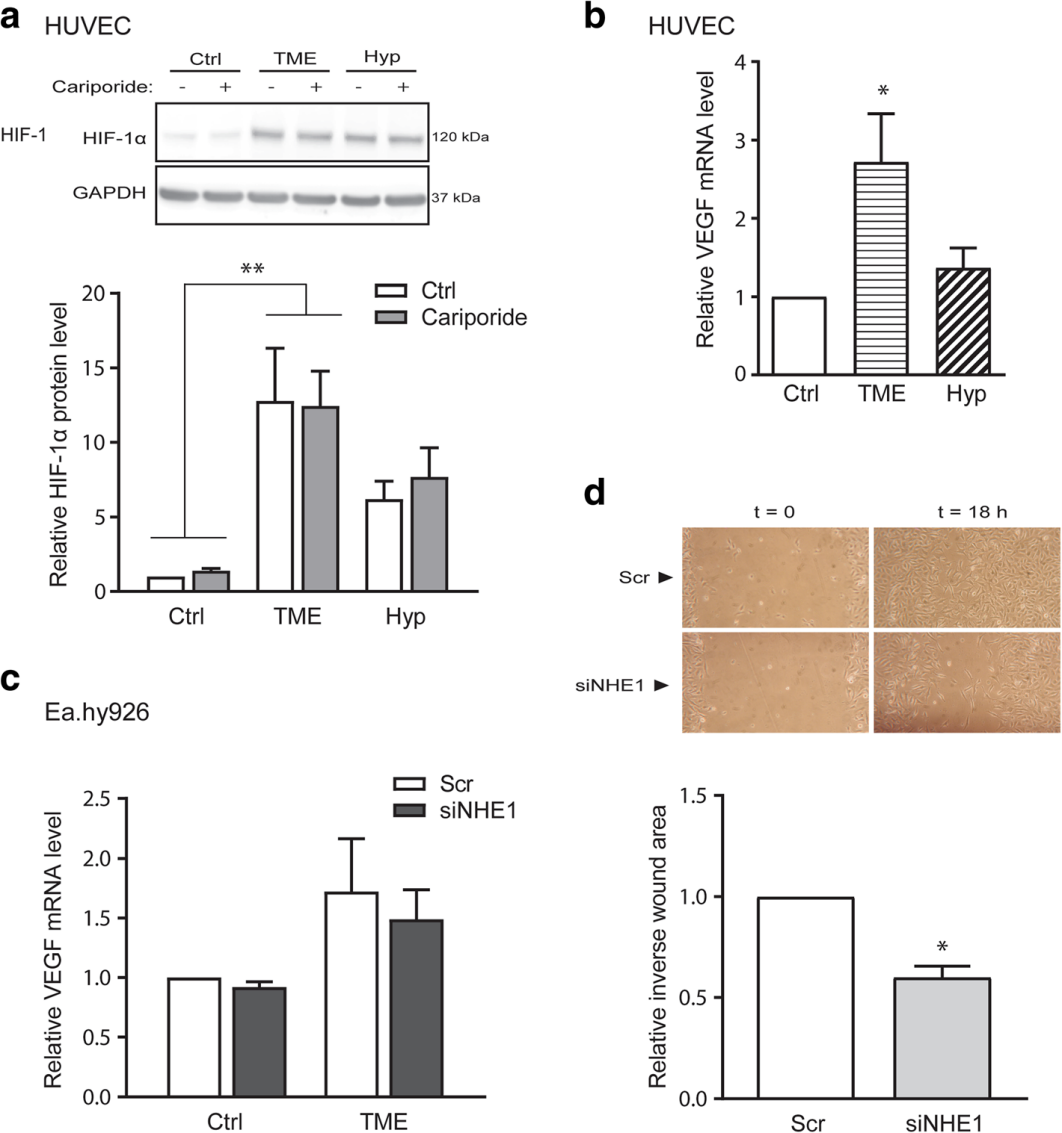
TME conditions upregulate HIF-1α and VEGF – this is NHE1-independent whereas endothelial cell migration is dependent on NHE1. HUVECs or Ea.hy926 were grown under normoxic control (Ctrl), simulated tumor microenvironment (TME; 1% O_2_, 1% FBS, 2.5 mM glucose, 7.5 mM lactate and pH 6.5) or hypoxic (Hyp; 1% O_2_) conditions for 24 h, prior to cell lysis and western blotting with primary antibodies against HIF-1α, or RNA purification, reverse transcription and qPCR with primers against VEGFA_165_. NHE1 was inhibited by cariporide (10 μM) or knocked down by siRNA treatment as indicated. **a** Representative western blot and quantification of HIF-1α protein levels after 24 h relative to the untreated control. GAPDH is shown as loading control. Quantified data are presented as means with SEM error bars of *n* = 3–5. ** indicate *p* < 0.01 compared to control cells, two-way ANOVA with Bonferroni’s multiple comparison post-test. The two-way ANOVA test also revealed a significant difference between conditions (Ctrl, TME, Hyp) with *p* < 0.0001. **b**, **c** Quantification of VEGF mRNA levels relative to the untreated control for HUVEC (B) and Ea.hy926 (C) cells. qPCR analysis was carried out as described in Methods, using GAPDH as housekeeping gene, and analysis was performed using the Pfaffl method. Data are shown as means with SEM error bars of *n* = 5. * denotes *p* < 0.05, one-way ANOVA with Tukey’s multiple comparison post-test. **d** Ea.hy926 cells were treated with NHE1 siRNA or scrambled control siRNA for 48 h (for knockdown efficacy, see Fig. 2d), whereafter a scratch in the culture was made with a sterile pipette tip and cell migration into the wound area monitored. Data are presented as means with SEM error bars of *n* = 3. The figure shows representative images and quantification of the wound area 18 h after scratch induction, relative to that of scrambled control siRNA

We first determined the effect of hypoxia and TME conditions on HIF-1α and VEGF levels. HUVEC, primary human endothelial cells, were exposed to either hypoxia alone (Hyp, 1% O_2_) or TME conditions (1% O_2_, 1% FBS, 2.5 mM glucose, 7.5 mM lactate, pH_e_ 6.5) for 24 h. Because of the proposed role of NHE1 in HIF-1α signaling in endothelial cells [25], we assessed the effect of these treatments in the absence and presence of NHE1 inhibition/knockdown. After exposure to hypoxia or TME for 24 h, HUVEC cells were lysed, followed by western blotting for HIF-1α (Fig. 1a). Notably, whereas hypoxia alone increased the HIF-1α protein level 7–8 fold compared to control conditions, TME exposure increased the HIF-1α level more than 12-fold. Inhibition of NHE1 by cariporide had no effect on the induction of HIF-1α expression in either condition (Fig. 1a). To determine whether the greater increase in HIF-1α level during TME compared to hypoxia was of functional significance, we assessed the mRNA level of VEGF in HUVEC after both conditions (Fig. 1b). Importantly, similar to the HIF-1α protein level, the VEGF mRNA level was increased much more by TME than by hypoxia alone. Consistent with this, the VEGF mRNA level in Ea.hy926 cells also tended to be increased after TME exposure, and this was unaffected by NHE1 knockdown under both control and TME conditions (Fig. 1c).

### NHE1 knockdown reduces endothelial cell migration

Given the central importance of NHE1 activity for migration in many cell types [39], we next asked whether knockdown of NHE1, rather than directly impacting HIF-1α signaling, affected endothelial cell migration. Confirming this hypothesis, NHE1 knockdown reduced wound closure of the Ea.hy926 cells at time 18 h after introduction of the wound, by about 40% (Fig. 1d).

Collectively, these results show that compared to hypoxia alone, TME conditions strongly potentiate HIF-1α accumulation, leading to an increased VEGF response. NHE1 inhibition or knockdown does not affect HIF-1α accumulation or VEGF production, but reduces endothelial cell migration, suggesting that reduced NHE1 levels in endothelial cells negatively impacts endothelial cell migration and hence angiogenesis in the TME.

### NHE1 is downregulated by hypoxia and TME conditions

**Fig. 2:**
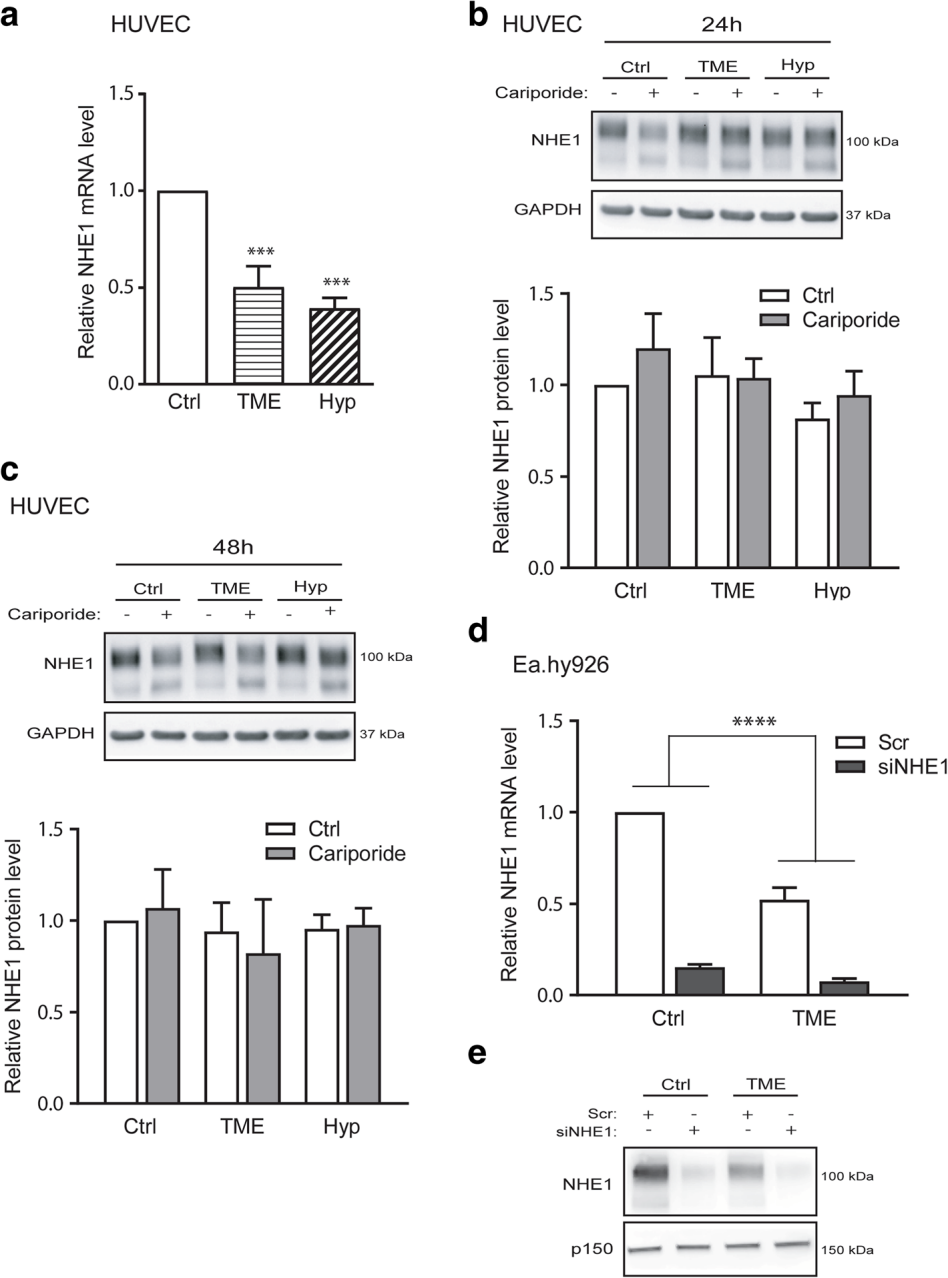
NHE1 is downregulated by hypoxia and TME. HUVECs or Ea.hy926 were grown under normoxic control (Ctrl), TME (1% O_2_, 1% FBS, 2.5 mM glucose, 7.5 mM lactate and pH 6.5) or hypoxic (Hyp; 1% O_2_) conditions for 24 h (or 48 h as indicated in panel C). Subsequently, cells were lysed and subjected to SDS-PAGE and western blotting with primary antibodies against NHE1 or RNA purification, reverse transcription and qPCR with primers against NHE1 and GAPDH, as described in the Methods section. NHE1 was inhibited by cariporide (10 μM) as indicated. **a** NHE1 mRNA levels in HUVEC based on quantification of qPCR results relative to the untreated control and normalized to GAPDH levels. *** indicates *p* < 0.001, ANOVA with Tukey’s multiple comparison post-test. Data are shown as means with SEM error bars of *n* = 5. **b** Representative western blot and quantification (relative to Ctrl conditions), showing the protein expression levels of NHE1 in HUVEC after 24 h of TME or hypoxia exposure. GAPDH is shown as loading control. Quantified data are shown as means with SEM error bars of *n* = 3–5. **c** Representative western blot and quantification (relative to ctrl condition), showing the protein expression levels of NHE1 in HUVEC after 48 h of TME or hypoxia exposure. GAPDH is shown as loading control. Quantified data are shown as means with SEM error bars of *n* = 3. **d**, **e** Effects of NHE1 siRNA knockdown and TME conditions were evaluated using the Ea.hy926 cell line. Cells were treated with siRNA against NHE1 or scrambled control siRNA for 24 h prior to exposure to TME conditions. **d** NHE1 mRNA levels in Ea.hy926 quantified as in (A). Data are shown as means with SEM error bars, and *n* = 5. **e** Western blot analysis of NHE1 protein levels in Ea.hy926. p150 is shown as loading control. Representative of *n* = 3

Having demonstrated an important role for NHE1 in endothelial cell migration, we next asked whether hypoxia and TME conditions altered NHE1 expression in endothelial cells. Notably, the mRNA level of NHE1 was significantly decreased by both TME conditions and by hypoxia alone (Fig. 2a), whereas these changes were not reflected at the NHE1 protein level in HUVEC, neither after 24 h (Fig. 2b) nor after 48 h (Fig. 2c). The specific NHE1 inhibitor cariporide (10 μM) had no effect on the NHE1 protein level in HUVEC under any of the conditions tested (Fig. 2b-c). To further pursue the effect of TME conditions on NHE1 expression in endothelial cells, we repeated these experiments in the endothelial hybrid cell line Ea.hy926. Similar to the finding in HUVEC, the NHE1 mRNA level was decreased by about 50% in Ea.hy926 cells after TME exposure (Fig. 2d), and in these cells, western blotting revealed a corresponding reduction in the NHE1 protein level after TME exposure (Fig. 2e). Transfection of Ea.hy926 cells with NHE1 siRNA strongly reduced both the mRNA (Fig. 2d) and protein (Fig. 2e) level of NHE1 under both control and TME conditions, compared to that in mock-transfected controls.

These results show that NHE1 mRNA expression was reduced by TME exposure in both endothelial cell types, whereas within the time course of this experiment, the NHE1 protein level was reduced in Ea.hy926 cells only.

### TME conditions strongly downregulate Akt in endothelial cells

**Fig. 3:**
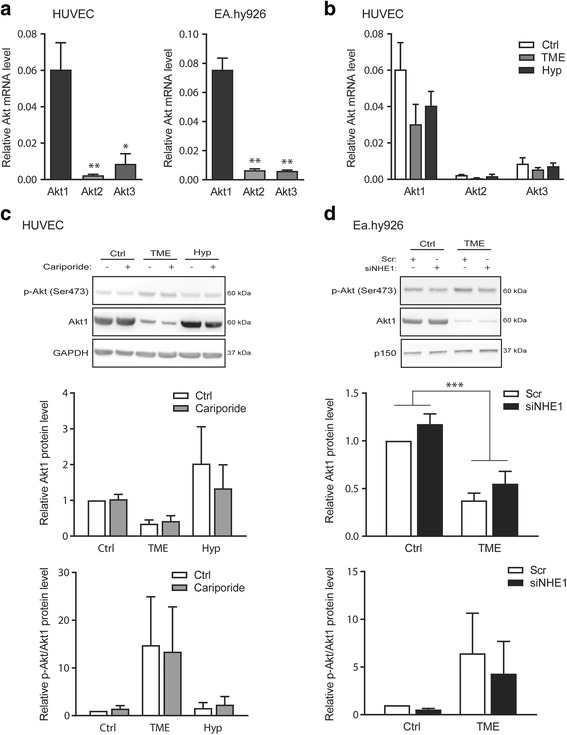
TME conditions dramatically lower mRNA and protein levels of Akt1 in HUVEC and Ea.hy926 cells, associated with increased relative phosphorylation of Akt. Cells were exposed to normoxic control (Ctrl), TME (1% O_2_, 1% FBS, 2.5 mM glucose, 7.5 mM lactate and pH 6.5) or hypoxic (Hyp; 1% O_2_) conditions as indicated, for 24 h before lysis and RNA purification, reverse transcription and qPCR or western blotting, as indicated. **a** Relative mRNA levels of the three Akt isoforms Akt1–3 in HUVEC (left panel) and Ea.hy926 (right panel) under Ctrl conditions. **b** Relative mRNA levels of Akt1–3 in HUVECs exposed to Ctrl, TME or Hyp conditions. Data are shown as means with SEM error bars and *n* = 5. * and ** denotes *p* < 0.05 and *p* < 0.01, respectively, one-way ANOVA with Tukey’s multiple comparison post-test. **c** Akt1 and p-Ser473Akt levels in HUVEC cells after 24 h of Ctrl, TME or Hyp conditions in the absence or presence of 10 μM cariporide. Top: representative western blots (GAPDH is shown as loading control), middle: protein level of total Akt1, bottom: p-Ser473Akt normalized to total Akt1. **d** As C, except for Ea.hy926 cells treated with NHE1 siRNA or scrambled control siRNA, and exposed to Ctrl or TME conditions. p150 is shown as loading control. Data are shown as means with SEM error bars, relative to control, and *n* = 3 for Hyp conditions, *n* = 5 for all other conditions. *** denotes *p* < 0.001, two-way ANOVA with Bonferroni’s multiple comparison post-test. The test revealed a significant difference in Akt1 protein levels between conditions (Ctrl, TME, Hyp), *p* < 0.01 for HUVEC and between conditions (Ctrl, TME), *p* < 0.0001 for Ea.hy926 cells

Given the central role of Akt in regulating endothelial cell function and the proposed role of NHE1 in regulation of Akt, we next asked how hypoxia, TME, and NHE1 inhibition/knockdown affected Akt expression and activity. Akt1 was the predominant Akt isoform in both HUVEC (Fig. 3a, left panel) and Ea.hy926 cells (Fig. 3a, right panel). Exposure to hypoxia or TME conditions decreased the mRNA level of Akt1 in HUVEC, by about 35% (hypoxia) and more than 50% (TME), respectively (Fig. 3b). Akt2 and −3 levels showed a similar pattern. In Ea.hy926 cells, TME downregulated Akt1 and −2 mRNA, but had no apparent effect on Akt3 (Additional file 1: Figure S1).

Also the protein level of Akt1 in HUVEC was potently decreased by TME exposure, whereas it was apparently unaffected by hypoxia alone (Fig. 3c). Akt activity was assessed by western blotting against Akt phosphorylated on Ser473 (p-Ser473Akt). Notably, it is evident from the representative blot in Fig. 3c that the p-Ser473Akt level increases, whereas total Akt1 decreases, in TME conditions, and that hypoxia alone has little effect. Data were quantified as total cellular Akt1 (middle) and p-Ser473Akt relative to total Akt1 (bottom). Inhibition of NHE1 by cariporide had no detectable effect on either Akt1 expression or Akt phosphorylation. Similarly, in Ea.hy926 cells (Fig. 3d), the total Akt1 level was decreased by about 50% after TME conditions, and the p-Ser473Akt/Akt1 ratio was increased by TME. Knockdown of NHE1 had no significant effect on the p-Ser473Akt/Akt1 ratio, consistent with the pharmacological data.

Taken together, these results show that the physicochemical TME exerts profound effects on Akt expression and activation not seen under hypoxia alone.

### Tumor cell conditioned medium exposure increases protein expression of Akt1 in HUVEC

**Fig. 4:**
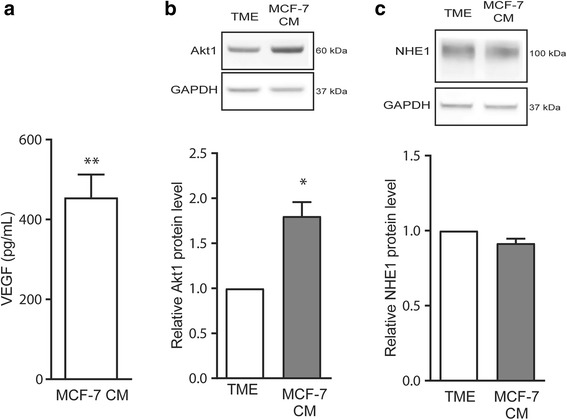
Treatment of HUVECs with tumor conditioned medium increases Akt, but not NHE1, protein expression. **a** VEGF content in MCF-7 conditioned medium (MCF-7 CM) based on quantification of ELISA results. MCF-7 cells were grown under TME conditions (1% O_2_, 1% FBS, 2.5 mM glucose, 7.5 mM lactate and pH 6.5) for 24 h, and medium collected for ELISA. Data are presented as mean with SEM error bar (*n* = 4). ** indicates *p* < 0.01, one-sample Student’s *t*-test against baseline. **b**, **c** HUVECs were exposed to standard TME conditions or to TME conditions with freshly prepared MCF-7 CM for 24 h followed by lysis and western blotting. Representative western blots and quantification of total protein levels relative to that for cells grown under standard TME conditions are shown for Akt1 (**b**) and NHE1 (**c**). GAPDH is shown as loading control. Data are presented as means ± SEM, with *n* = 4 per condition. * indicates *p* < 0.05, two-tailed paired Student’s *t*-test

In the in vivo tumor microenvironment, cancer cells and stromal cells secrete VEGF and other cytokines and growth factors that impact endothelial cell function [8]. Consistent with this notion, MCF-7 human breast cancer cells grown under TME conditions secreted large amounts of VEGF, which was detectable in the medium by ELISA (Fig. 4a). In contrast, VEGF protein was not detectable in HUVEC medium under these conditions (not shown), although VEGF mRNA was readily detected in HUVEC lysates (Fig. 1b). We therefore speculated that the secretome from the cancer cells might counteract the repression of endothelial Akt induced by the physicochemical TME conditions. To address this, we exposed HUVEC to conditioned medium from MCF-7 cells, generated under TME conditions identical to those used for the endothelial cells. Notably, exposure to this MCF-7 tumor conditioned medium (MCF-7 CM) resulted in a nearly 2-fold increase in Akt1 protein in HUVEC relative to regular TME conditions (Fig. 4b), whereas it had no effect on NHE1 expression (Fig. 4c).

In conjunction with the TME-induced VEGF mRNA increase in the endothelial cells shown in Fig. 1 and the reduction in Akt levels by TME shown in Fig. 3, this result suggests that although endothelial cells do increase VEGF production under TME conditions, additional paracrine stimulation from the cancer cells is important for endothelial cells to maintain Akt expression under these conditions.

### TME conditions, but not hypoxia, decrease signaling related to protein synthesis

**Fig. 5:**
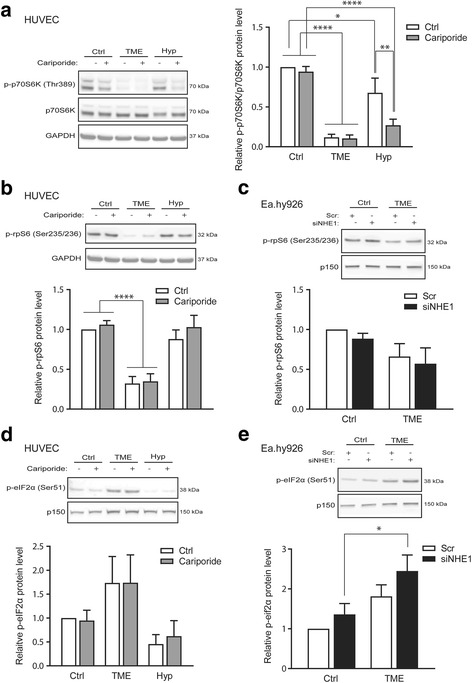
TME conditions regulate the phosphorylation levels of p70S6K, rpS6 and eIF2α. HUVECs or Ea.hy926 were exposed to normoxic control (Ctrl), TME (1% O_2_, 1% FBS, 2.5 mM glucose, 7.5 mM lactate and pH 6.5) or hypoxic (Hyp; 1% O_2_) conditions as indicated, followed by lysis and western blotting. NHE1 was inhibited by cariporide (10 μM) or knocked down by siRNA-treatment where indicated. **a** Representative western blots of p-Thr389p70S6K and total p70S6K and quantification of p-p70S6K protein levels normalized to total p70S6K and relative to the untreated control condition for 24 h for HUVEC. GAPDH is shown as a loading control. **b**, **c** Representative western blots of p-Ser235/236rpS6 and quantifications of p-rpS6 protein levels relative to the untreated control condition for 24 h for HUVEC (**b**) and Ea.hy926 (**c**). GAPDH and p150 are shown as loading controls. **d**, **e** Representative western blots of p-Ser51eIF2α and quantifications of p-eIF2α protein levels relative to the untreated control condition for 24 h for HUVEC (**d**) and Ea.hy926 (**e**). p150 is shown as loading control. Data are presented as means with SEM error bars, with *n* = 5 except Hyp without/with cariporide for which *n* = 3. *, ** and *** indicates *p* < 0.05, *p* < 0.01 and *p* < 0.001, respectively, as obtained by two-way ANOVA with Bonferroni’s multiple comparison post-test. The two-way ANOVA test revealed a significant difference in p-p70S6K/p70S6K (*p* < 0.0001), p-rpS6 (*p* < 0.0001 for HUVEC and *p* < 0.05 for Ea.hy926) and p-eIF2α (*p* < 0.05 for HUVEC and *p* < 0.01 for Ea.hy926) between conditions (Ctrl, TME, Hyp). Also, p-p70S6K/p70S6K significantly changed with cariporide (*p* < 0.01)

An important downstream effect of Akt signaling is endothelial cell growth, a process mediated in large part through increased translation via phosphorylation and activation of p70S6K and its substrate the ribosomal protein rpS6 [29, 33]. Western blotting showed that the level of Thr389-phosphorylated, active p70S6K was dramatically decreased under TME conditions in HUVEC (Fig. 5a). Hypoxia alone also reduced p70S6K phosphorylation, although to a much lesser extent. Interestingly, NHE1 inhibition by cariporide further reduced the hypoxic level of Thr389-p70S6K. rpS6 is a target of p70S6K, and accordingly, the Ser235/236-phosphorylation of rpS6 was substantially reduced in HUVEC under TME conditions, whereas it was unaffected by hypoxia alone (Fig. 5b). A similar trend was seen after TME exposure in Ea.hy926 cells (Fig. 5c). rpS6 phosphorylation was unaffected by cariporide in both cell lines (Fig. 5b, c). Whereas rpS6 phosphorylation by the Akt - p70S6K pathway correlates with increased protein translation, phosphorylation of the translation initiation factor eIF2α at Ser51 by one of several eIF2α kinases reduces the rate of protein translation by stabilizing the GDP bound state of eIF2α, rendering it inactive [40]. Notably, in HUVEC, the phosphorylation level of eIF2α was increased by TME exposure, yet unaffected by hypoxia alone, consistent with reduced protein translation (Fig. 5d), and a similar pattern was seen in Ea.hy926 cells (Fig. 5e). The phosphorylation state of eIF2α was insensitive to cariporide in both cell types (Fig. 5d, e).

These results reveal a remarkable difference between the impact of TME and of hypoxia alone on translation in endothelial cells: several branches of the translational machinery are negatively regulated by TME conditions, yet unaffected by hypoxia alone.

### TME, but not hypoxia, reduces proliferation and induces apoptosis signaling in endothelial cells

**Fig. 6:**
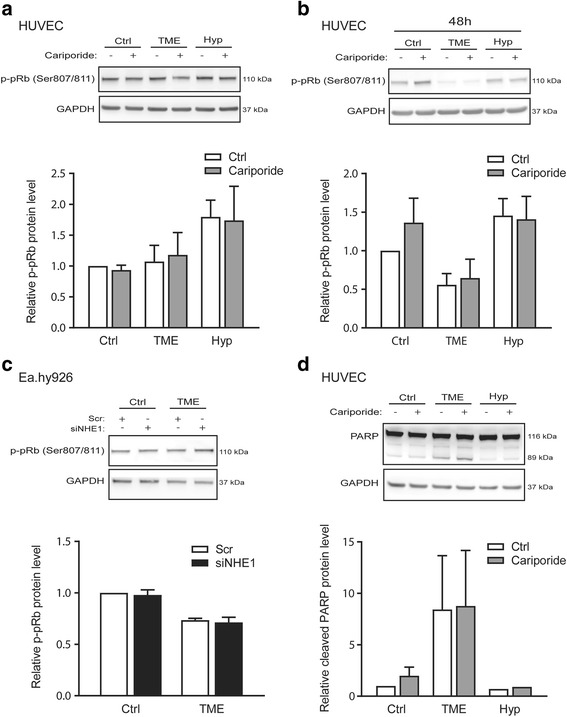
TME conditions decrease proliferation and increase apoptosis signaling, while hypoxia alone has no effect. HUVECs or Ea.hy926 were exposed to normoxic control (Ctrl), TME (1% O_2_, 1% FBS, 2.5 mM glucose, 7.5 mM lactate and pH 6.5) or hypoxic (Hyp; 1% O_2_) conditions as indicated, for 24 or 48 h followed by lysis and western blotting. NHE1 was inhibited by cariporide (10 μM) or knocked down by siRNA-treatment where indicated. GAPDH is shown as loading control. **a**, **b**, **c** Representative western blots of p-Ser807/811-pRb and quantifications of the p-pRb protein levels relative to the untreated control condition for 24/48 h for HUVEC (**a**, **b**) and 24 h for Ea.hy926 (**c**). Two-way ANOVA revealed a significant difference in p-pRb levels between conditions (Ctrl, TME, Hyp) with *p* < 0.05 for HUVEC and *p* < 0.01 for Ea.hy926, respectively. **d** Representative western blots showing PARP protein levels and quantification of cleaved PARP relative to the untreated control for 24 h in HUVEC. Data are presented as means with SEM error bars, with *n* = 3–5

We next asked whether the much more potent effects of TME conditions compared to hypoxia alone would translate into different effects of the two conditions on endothelial cell proliferation and survival. We first determined the effects of TME and hypoxia on proliferation signaling in HUVEC and Ea.hy926 cells, using p-Ser807/811-retinoblastoma protein (p-pRb) as a marker of the level of cell proliferation (Ser807/811 phosphorylation of pRb allows G1 progression [41]). In HUVEC, neither TME nor hypoxia markedly affected pRb phosphorylation after 24 h of exposure (Fig. 6a), but after 48 h, TME conditions had induced a ~ 50% decrease in the p-pRb level, whereas there was no effect of hypoxia (Fig. 6b). Ea.hy926 cells responded more rapidly to TME conditions, with a decreased p-pRb level detectable after 24 h of TME exposure (Fig. 6c). There was no effect of either cariporide (Fig. 6a, b) or NHE1 knockdown (Fig. 6c) on pRb phosphorylation.

To evaluate apoptotic cell death induction, we evaluated the appearance of the 89 kDa cleavage product of poly-ADP-ribose polymerase (PARP) [42]. HUVEC exhibited a marked increase in PARP cleavage after 24 h of TME conditions, whereas neither hypoxia alone nor cariporide treatment affected PARP cleavage (Fig. 6d).

Taken together, these data indicate that TME exposure, but neither hypoxia nor NHE1 inhibition, reduces proliferation and induces apoptosis in endothelial cells.

## Discussion

In the great majority of cancers, neo-vascularization is essential for continued tumor growth [1–3]. Hypoxia is only one component of the TME, which is additionally characterized by low levels of nutrients and glucose, elevated lactate, and low pH_e_, and the presence of VEGF and numerous other cytokines and growth factors [8]. Although it is well established that these other TME components can exert a profoundly different gene-regulatory effect than hypoxia alone [9–12] very few studies have directly compared the effects of hypoxia versus a more complex TME on angiogenic signalling. The Na^+^/H^+^ exchanger NHE1 plays a major role in cancer development [14, 15], is regulated by hypoxia [27, 43] and was recently proposed to play a role in HIF-1α-induced angiogenesis [25], yet its roles in endothelial cell signaling under hypoxia as compared to TME conditions have not been studied. The aims of this work were, therefore, to investigate the effects of a complex TME versus hypoxia alone on two different endothelial cell lines and to evaluate the possible roles of NHE1 in the endothelial cell response to TME and hypoxia.

### HIF-1α and VEGF are induced more potently by TME than by hypoxia alone

It is well established that HIF-1α activation is induced in response to hypoxia in endothelial cells, and that HIF-1α mediates increased expression of VEGF and other angiogenic factors leading to endothelial cell activation [44]. We show here that TME had a much stronger effect on HIF-1α and VEGF expression than hypoxia alone, demonstrating that other components of the TME potentiate this response. A very likely candidate is lactate, which was previously found to induce HIF-1α protein expression and activity in HUVEC. The mechanism involves lactate uptake via monocarboxylate transporter 1 (MCT1) conversion to pyruvate, and inhibition of prolyl hydroxylase 2 (PHD2), preventing HIF-1α degradation [45, 46]. A lactate-induced increase in released VEGF from HUVEC was not detectable [46]. In contrast, lactate elicited upregulation of VEGFR2, which would potentiate signaling by VEGF released by the cancer cells in a tumor [45, 46]. Interestingly, VEGF mRNA levels in HUVEC were increased after 48 h exposure to 10 mM lactate [47], consistent with our finding after TME exposure. Collectively, this suggests that mRNA levels of VEGF in HUVEC are modestly increased by lactate as well as by TME, yet do not elicit detectable VEGF release, in contrast to the robust VEGF release measured from TME-treated MCF-7 breast cancer cells.

Another possible effector is acidic pH_e_, which has been shown to increase HIF-1α stability in several cell types, by mechanisms proposed to involve repression of the PHD2/von Hippel-Lindau (VHL) pathway [48] or a heat shock protein 90 (HSP90)-dependent pathway [49]. In other cell types no or only a slight enhancement of HIF-1α expression was reported [50]. Low pH_e_ was also shown to increase VEGF production in glioblastoma cells in a HIF-1α –independent, ERK1/2/AP-1-dependent manner [51].

NHE1 inhibition or knockdown had marginal or no effect on the other studied signaling events elicited by TME or hypoxia (discussed in the following sections). This was initially unexpected as a 0.2–0.3 pH unit decrease in HUVEC pH_i_ after NHE1 knockdown was previously reported [25]. However, these measurements were carried out in PBS solution, preventing pH_i_ regulation by the Na^+^, HCO_3_^−^-cotransporters which are known to play a major role in this process in most endothelial cells [14], and hence are unlikely to reflect the physiological situation.

Thus, we conclude that TME robustly upregulates HIF-1α and VEGF expression in endothelial cells in a manner that may involve effects of lactate and/or acidic pH_e_ in addition to hypoxia. Further, we conclude that in the cell types studied here, this upregulation does not involve NHE1.

### NHE1 is downregulated by TME and hypoxia, and its knockdown reduces endothelial cell migration but has no effect on other TME-induced signaling events

In contrast to the increase in HIF-1α and VEGF, the mRNA level of NHE1 was strongly reduced by both TME and hypoxia conditions. The NHE1 protein level also decreased in Ea.hy296 cells after 24 h of TME, but not in HUVEC over 48 h. This may be related to slower protein degradation (basal or TME induced) in HUVEC, the reported half-time for NHE1 in the membrane under normal conditions being about 24 h as measured in fibroblasts and epithelial cells [52]. The reduction of NHE1 protein expression by hypoxia is consistent with recent work in which 48 h of 2% hypoxia or exposure to the HIF-1α stabilizer Dimethyloxaloylglycine (DMOG) reduced NHE1 protein levels in some cancer cell lines [27]. In contrast, NHE1 was upregulated in cerebral microvascular endothelial cells (CMEC) exposed for 1–5 h to hypoxia [24] and in pulmonary arterial myocytes after 3 weeks at 10% O_2_ [26], suggesting dose- and cell-type-dependent differences in the effects of hypoxia on NHE1 expression. Interestingly, in CMEC, upregulation of NHE1 by hypoxia was greater at 7% than at 2% oxygen [24], hinting that the cell type differences may involve a balance between hypoxia-induced signals favoring NHE1 up- and downregulation. Importantly, it is clear that the more physiologically relevant TME and hypoxia conditions used here do not recapitulate the reported upregulation of NHE1 in HUVEC after lentiviral HIF-1α overexpression [25].

Importantly, consistent with the known role of NHE1 in the motility of a wide range of cell types [39], knockdown of NHE1 in Ea.hy926 cells – i.e. resembling the effect of TME on these cells – reduced their motility in a scratch wound assay by 50%. The observed reduction in the NHE1 level under TME conditions is therefore likely to result in reduced endothelial cell motility under these conditions.

### Akt and its downstream effectors are regulated by TME but not by hypoxia

Consistent with earlier reports on Akt isoform expression in endothelial cells, [32] HUVECs expressed Akt1, −2, and −3, yet Akt1 was by far the most abundant [32]. The decrease in Akt expression under TME conditions was seen both at the mRNA and protein levels, whereas hypoxia alone modestly decreased the Akt1 mRNA level, but had no effect on the protein level. This suggests that Akt protein stability may be decreased by factors in the TME. The mechanisms, although not further studied here, could include the low serum content of TME medium, as it has been shown that serum deprivation downregulated Akt in HUVEC [53]. Supporting our finding that the VEGF-enriched, cancer cell conditioned medium rescued Akt1 expression, the same study showed that culturing cells with VEGF prevented downregulation of Akt in response to serum deprivation [53]. Also in congruence with our findings, tumor conditioned medium was shown to increase Akt activity in microvascular endothelial cells in a VEGFR2 dependent manner [54].

Akt positively regulates cell growth and protein synthesis through activation of mTORC1 and downstream activation of the p70S6 kinase and regulation of downstream substrates rpS6 and eIF4B [29, 33, 40]. TME elicited reduced phosphorylation of both p70S6K and rpS6, which is in accordance with the reduced total Akt level, but surprising given that the total Akt phosphorylation was not reduced by TME. The underlying mechanisms are not clear, but an isoform shift in Akt activity is possible, since the different Akt isoforms have different downstream effects [30–32]. Furthermore, as lack of nutrients and energy (low levels of growth factors, glucose, amino acids and oxygen) results in inhibition of mTORC via complex signaling events not only downstream of Akt [33], this could in principle contribute to the observed effects of TME on p70S6K and consequently rpS6. Although the precise mechanisms mediating the phosphorylation of eIF2α under TME conditions remain to be determined, eIF2α phosphorylation is induced by several different kinases under stress conditions, including endoplasmic reticulum (ER) stress [40]. Notably, mechanisms have evolved for specific transcripts to evade general inhibition of protein synthesis as under TME conditions. These mechanisms are often exploited to induce translation of adaptive proteins under conditions of cellular stress. An example is the presence of internal ribosome entry sites (IRESs), which are present in ~10% of all mRNAs and allow translation when regular cap-dependent translation is inhibited. VEGF contains two IRESs [55, 56] and the presence of these could mediate expression of VEGF under TME conditions even though global translation was inhibited.

### Implications for endothelial cell function in the tumor microenvironment in vivo

TME treatment reduced proliferation signaling and induced PARP cleavage in the endothelial cells, a finding supported by earlier reports that HUVEC undergo caspase-dependent death upon deprivation of serum/nutrients [28, 53], and by a recent report showing that HUVEC proliferation is reduced by acidic pH_e_ [57]. The results also fit well with the downregulation of Akt, which normally favors cell proliferation and counteracts cell death via phosphorylation-mediated regulation of e.g. GSK3 and BAD [33]. It is surprising that NHE1 knockdown does not further exacerbate these effects, given the known roles of this transporter in growth and survival under stress conditions [58], however, it may simply be that knockdown has no further effect on proliferation and death because of the already decreased NHE1 levels under TME conditions. It is possible that similar to Akt (but not NHE1) expression, the effects of TME on proliferation and survival are at least partially compensated in vivo, e.g. by VEGF and survival factors released from the cancer cells and other stromal cells [8]. Given their markedly different genetic profiles [59, 60], it also seems likely that TME elicits different signaling events in tumor endothelial cells than in normal endothelial cells, and this should be assessed in future studies. It is known, for instance, that endothelial cells at the forefronts of sprouts are capable of adapting to severe hypoxia, since most of their energy is obtained through anaerobic glycolysis [61, 62]. In addition, microvascular (but not macrovascular) endothelial cells express vacuolar-type H^+^-ATPases at the membrane [63], and could thus be able to maintain a relatively normal pH_i_ during periods of increased glycolysis, as in the hypoxic microenvironment of tumors.

## Conclusions

In conclusion, we show here that TME conditions upregulate HIF-1α and VEGF, and downregulate NHE1 and Akt, in endothelial cells, much more potently than does hypoxia alone. The downregulation of Akt can partially be compensated by cancer cell conditioned medium. Likely downstream from the decrease in Akt expression, TME exposure elicits reduced p70S6K and rpS6 phosphorylation and increases eIF2α phosphorylation, consistent with reduced protein translation. Finally, TME elicits reduced proliferation and increased apoptosis signaling. These effects are independent of NHE1, yet knockdown of NHE1 to levels similar to those induced by TME strongly reduces endothelial cell migration.

## Additional file

Additional file 1: Figure S1. TME downregulates Akt1 and −2, but not −3 mRNA levels in Ea.hy926 cells. Cells were exposed to the normoxic control (Ctrl), or TME (1% O_2_, 1% FBS, 2.5 mM glucose, 7.5 mM lactate and pH 6.5) conditions for 24 h before lysis and RNA purification, reverse transcription and qPCR. The graphs show the relative mRNA expression levels of the three Akt isoforms Akt1–3 in Ea.hy926 exposed to Ctrl, or TME conditions and treated with siRNA targeting NHE1 as indicated. Results were based on quantification of qPCR results obtained using specific primers against Akt1, −2 or −3 and normalized to GAPDH. Data are shown as means with SEM error bars and *n* = 5. ** and *** denotes *p* < 0.01 and *p* < 0.001, respectively, acquired using two-way ANOVA with Bonferroni’s multiple comparison post-test. (TIFF 1715 kb)

## Abbreviations

BAD: Bcl-2 antagonist of death
CMEC: Cerebral microvascular endothelial cells
DMOG: Dimethyloxaloylglycine
eIF2α: Eukaryotic Initiation Factor 2
ERK1/2: Extracellular signal regulated kinase 1/2
GSK3: Glycogen synthase kinase 3
HIF-1α: Hypoxia-inducible factor-1α
HUVEC: Human umbilical vein endothelial cells
IRES: Internal ribosome entry site
MCT1: Monocarboxylate transporter 1
mTORC: Mammalian target of rapamycin complex-1
NHE1: Na^+^/H^+^ exchanger 1 p70S6K p70S6 kinase
PHD2: Prolyl hydroxylase 2
pH_e_: Extracellular pH
pH_i_: Intracellular pH
PARP: Poly-ADP-ribose polymerase
rpS6: Ribosomal protein
TME: Tumor microenvironment
VEGF: Vascular endothelial growth factor
VEGFR2: VEGF receptor 2
VHL: von Hippel-Lindau
