# Supplementary figures and images for: Tumor microenvironment conditions alter Akt and Na+/H+ exchanger NHE1 expression in endothelial cells more than hypoxia alone: implications for endothelial cell function in cancer
Source: BMC Cancer. 2017 Aug 14;17:542. doi: 10.1186/s12885-017-3532-x (PMC5556346; doi:10.1186/s12885-017-3532-x)

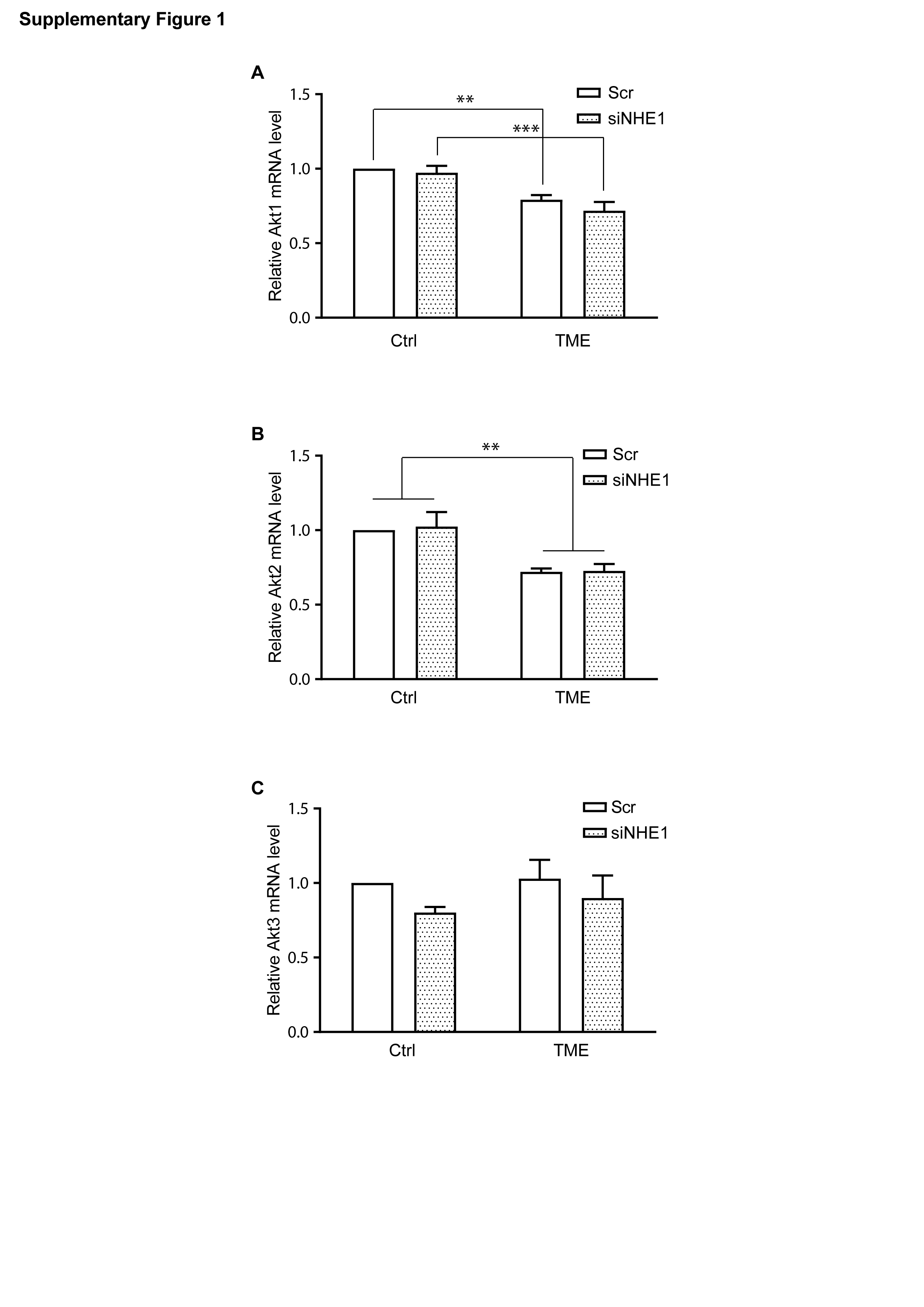

Supplement: Additional file 1: Figure S1. — TME downregulates Akt1 and −2, but not −3 mRNA levels in Ea.hy926 cells. Cells were exposed to the normoxic control (Ctrl), or TME (1% O2, 1% FBS, 2.5 mM glucose, 7.5 mM lactate and pH 6.5) conditions for 24 h before lysis and RNA purification, reverse transcription and qPCR. The graphs show the relative mRNA expression levels of the three Akt isoforms Akt1–3 in Ea.hy926 exposed to Ctrl, or TME conditions and treated with siRNA targeting NHE1 as indicated. Results were based on quantification of qPCR results obtained using specific primers against Akt1, −2 or −3 and normalized to GAPDH. Data are shown as means with SEM error bars and n = 5. ** and *** denotes p < 0.01 and p < 0.001, respectively, acquired using two-way ANOVA with Bonferroni’s multiple comparison post-test. (TIFF 1715 kb) [file 12885_2017_3532_MOESM1_ESM.tif]
